# A Morrison stem gekkotan reveals gecko evolution and Jurassic biogeography

**DOI:** 10.1098/rspb.2023.2284

**Published:** 2023-11-29

**Authors:** Dalton Meyer, Chase D. Brownstein, Kelsey M. Jenkins, Jacques A. Gauthier

**Affiliations:** ^1^ Department of Earth and Planetary Sciences, Yale University, New Haven, CT 06506520-8109, USA; ^2^ Department of Ecology and Evolutionary Biology, Yale University, New Haven, CT 06520-8106, USA; ^3^ Stamford Museum and Nature Center, Stamford, CT 06903, USA; ^4^ Yale Peabody Museum, New Haven, CT 06520-8118, USA

**Keywords:** *Squamata*, *Gekkota*, phylogenetics, macroevolution, biogeography

## Abstract

Geckos are a speciose and globally distributed clade of *Squamata* (lizards, including snakes and amphisbaenians) that are characterized by a host of modifications for nocturnal, scansorial and insectivorous ecologies. They are among the oldest divergences in the lizard crown, so understanding the origin of geckoes (*Gekkota*) is essential to understanding the origin of *Squamata*, the most species-rich extant tetrapod clade. However, the poor fossil record of gekkotans has obscured the sequence and timing of the assembly of their distinctive morphology. Here, we describe the first North American stem gekkotan based on a three-dimensionally preserved skull from the Morrison Formation of western North America. Despite its Late Jurassic age, the new species already possesses several key characteristics of the gekkotan skull along with retained ancestral features. We show that this new stem gekkotan, and several previously named species of uncertain phylogenetic relationships, comprise a widespread clade of early crown lizards, substantiating faunal homogeneity in Laurasia during the Late Jurassic that extended across disparate ecological, body-size and physiological classes.

## 1. Introduction

*Gekkota* is a charismatic, speciose, disparate and widely distributed [1] clade of lizards routinely rooting near the base of the lizard tree [2,3]. There are, however, persistent conflicts regarding the position of the root of the squamate crown inferred from molecular versus morphological data [4,5]. Molecular analyses have yet to convincingly resolve relationships among gekkotans, dibamids and unidentatans (all other lizards) that they place at the base of *Squamata* [2,6]. By contrast, morphological trees [3,7] firmly root between *Iguania* and the rest of squamates (*Scleroglossa*), including gekkotans, which are the next crown divergence among scleroglossans. To further complicate matters, molecular analyses never find *Iguania* as sister to the rest of crown squamates [2]. Despite these complications, the relationships of *Gekkota* are clearly near the base of the major ‘backbone’ divergences in the lizard crown. Thus, a deeper understanding of gekkotan phylogeny will be vital to resolving the origin of *Squamata*. Because fossils improve morphological studies [8], the fossil record of *Pan-Gekkota,* the gecko total clade [9], is expected to play a central role in resolving these conflicts. Unfortunately, that record is poor, particularly in the Mesozoic [10], when most of the squamate backbone clades originated [2,11–13].

Here, we use high-resolution computed tomography and an updated morphological dataset to resolve the identity of a partial skull (DINO 15914) from the Late Jurassic Morrison Formation of Utah, USA, that was initially reported as a specimen of the basal pan-scincoid †*Paramacellodus cf. P. oweni* [14]. The anatomy of the new species, which is found to be the oldest pan-gekkotan from the Americas, reveals the complex history of trait acquisition in stem gekkotans that resulted in the highly modified skulls of extant species. The close relationship of the new stem gekkotan to other fossil squamates from Europe demonstrates that faunal continuity has existed at multiple levels among terrestrial vertebrate communities across the Northern Hemisphere during the Late Jurassic. The new species also pushes back the first occurrence of pan-gekkotans in North America by at least 100 million years (from the Eocene to the latest Jurassic) and shows that there was more than one dispersal of pan-gekkotans to the continent, with the earliest clade (*Ardeosauridae*) going extinct well before the arrival of any members of the crown (*Gekkota*). This highlights the importance and utility of fossils in generating and testing hypotheses of historical biogeography.

## 2. Results

### (a) Systematic paleontology

*Squamata* Oppel 1811 (*sensu* de Queiroz and Gauthier, 2020)

*Pan-Gekkota* Bauer 2020

†*Ardeosauridae* Camp 1923 [15] (see Taxonomic Notes in the Electronic Supplementary Information for the corresponding clade-name definition according to the International Code of Phylogenetic Nomenclature [16]).

†*Helioscopos dickersonae* gen. et sp. nov.

**Etymology.** *Helioscopos*, after the Ancient Greek *helios* (sun) and the Latinization of the Ancient Greek *scopós* (watcher) after the prominent pineal foramen found in this species, and *dickersonae,* the Latinized genitive form of ‘Dickerson’, in honour of Helen and Shirley Dickerson, the late grandmother and great aunt, respectively, of DM, as well as Mary Cynthia Dickerson, the first Curator of Herpetology and mentor to the celebrated lizard systematist Charles L. Camp, at the American Museum of Natural History.

**Figure 1.**
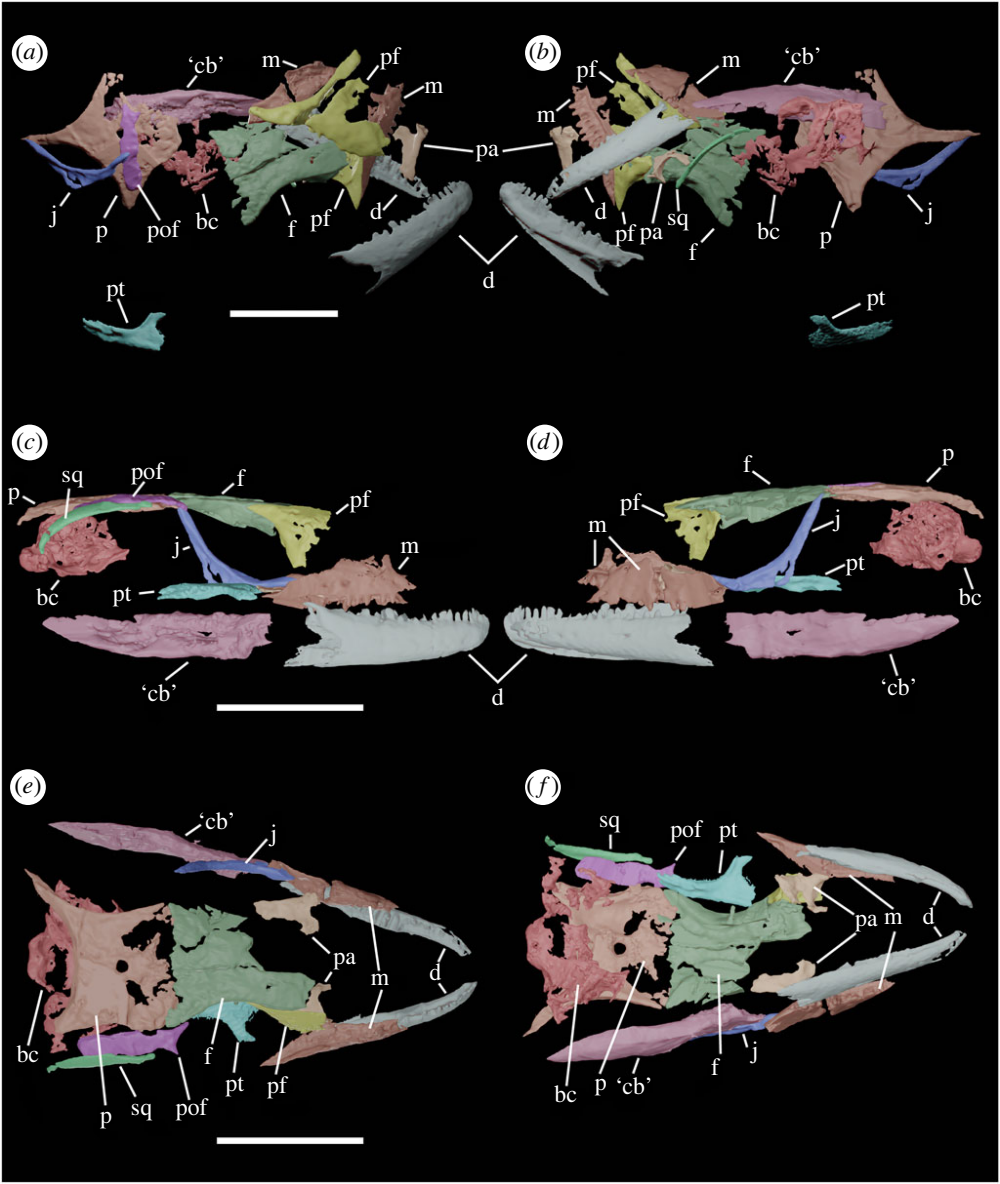
CT Imagery of the skull of *Helioscopos dickersonae* (DINO 15914). (*a,b*) *In situ* skull in dorsal (*a*) and ventral (*b*) views. (*c–f*) Reconstructed skull in left lateral (*c*), right lateral (*d*), dorsal (*e*) and ventral (*f*) views. All scale bars 5 mm. bc, braincase; ‘cb’, fused postdentary elements (compound bone); d, dentary; f, frontal; j, jugal; m, maxilla; p, parietal; pa, palatine; pf, prefrontal; pof, postorbitofrontal; pt, pterygoid; sq, squamosal.

**Holotype.** DINO 15914, partial skull and mandibles lacking the anterior snout (figure 1). Preserved elements include the maxillae, prefrontals, parietal, right postorbitofrontal and squamosal, left jugal, partial palatines and right pterygoid, partial braincase, dentaries and the fused left postdentary bones.

**Locality and horizon.** Dinosaur National Monument (DINO) site #317, Uintah County, Utah (electronic supplementary material, figure S6). Morrison Formation, Brushy Basin Member, Late Jurassic, Tithonian (149–150 Ma) [17].

**Comments on ontogenetic stage.** Because all characters differentiate during ontogeny, it is important to establish the developmental stage of a fossil individual to distinguish ontogenetic from phylogenetic sources for the apparent absence of any given apomorphy. To this end, we follow Griffin *et al*. [18] by providing this section discussing our ontogenetic assessment of DINO 15914. Two elements of ontogenetic significance are preserved in DINO 15914: the braincase and the posterior mandibular bones. Complete fusion of the braincase is a marker of skeletal maturity in squamates [19,20]. The braincase of DINO 15914 includes the contact between the sphenoid and basioccipital, and it is fused indistinguishably. The surangular and prearticular are also fused in DINO 15914. This is commonly seen across the (mostly) ontogenetically mature sample of squamates in Gauthier *et al*. [3] and is unambiguously indicative of skeletal maturity and age in at least one squamate species, *Aspidoscelis tigris* [20]. Based on this evidence, we infer that DINO 15914 was skeletally mature at death.

**Differential diagnosis.** Late Jurassic pan-gekkotan that differs from all other squamates in the following combination of features: Fused postorbitofrontal; frontoparietal suture is transversely straight (rather than bowed anteriorly at its midpoint as in †*Norellius nyctisaurops*); prominent pineal foramen (contra that of †*Ardeosaurus brevipes*); parietal nuchal fossa mediolaterally widens to more than two-thirds of the posterior margin of the parietal; midline posterior parietal projection absent; slender posterior projection of the parietal supratemporal process; palatines strongly bowed over the choanal fossa; palate edentulous. Differs from †*A. brevipes* in having a relatively larger pineal foramen; a fused postorbitofrontal; a more gracile jugal; a narrower anterior squamosal; lacking a midline postparietal projection; lacking any sculpturing indicating cephalic osteoderms. Differs from †'*Ardeosaurus*’ *digitatellus* in having wider separation between the supratemporal processes of the parietal; narrower anterior squamosals; greater curvature of the lateral parietal margins; wider frontals (relative to the frontoparietal suture). Differs from contemporary †*Eoscincus ornatus* in having an edentulous palate; frontal subolfactory processes that approach one another on the midline; a lack of rugosity on the dorsal surface of the frontals; more sharply constricted frontals; a more sharply rising narial margin of the maxilla; a longer suborbital ramus of the maxilla; a more gracile jugal; a broader opening of the choanal fossa of the palatine; more sharply ascending dentary coronoid process. Differs from contemporary †*Microteras borealis* in having a more sharply rising narial margin of the maxilla; a more prominent palatal shelf of the maxilla; a taller lateral aperture of the recessus scalae tympani (LARST) and concomitantly more vertically oriented cristae interfenestralis and tuberalis; occipital condyle with less pronounced midline concavity (in dorsal view). Differs from contemporary †*Diablophis gilmorei* in having taller maxillary facial process with more sharply angled narial margin; dentition lacking an expanded base; dentition not recurved. Differs from contemporary †*Dorsetisaurus* sp. in having: more closely packed dentition; lacking laterally compressed teeth; a more sharply rising narial margin of the maxilla.

**Diagnosis of genus.** As for the type species.

### (b) Description

The frontals of †*Helioscopos dickersonae* are paired and moderately constricted at their midpoint, a feature shared with all other members of †*Ardeosauridae* (figure 1) (as well as with several other groups of lizards). The frontal displays a prominent subolfactory process that arches ventromedially toward its contralateral process as in all other pan-gekkotans, but fails to meet it on the midline as in crown-gekkotans (electronic supplementary material, figure S1) [3,21,22]. Anteriorly, a small facet for the nasal is visible, and laterally there is an anterior facet for the prefrontal and a posterior facet for the fused postorbitofrontal (electronic supplementary material, figure S1). The postorbital and postfrontal are fused into a postorbitofrontal as in †‘*Ardeosaurus*’ *digitatellus* (see the following section for a new generic assignment of this species). It is anteroposteriorly elongate, with tapering frontal and jugal processes, while the posterior squamosal process of the postorbitofrontal is broad and plate-like and slightly bowed dorsally (figure 1), a feature that it shares with †*Ardeosaurus brevipes*.

The parietal is a broad and flat bone that overlies the brain posterodorsally. The parietal table is nearly as anteroposteriorly long as mediolaterally wide (figure 1) and the dorsal surface is smooth, lacking any indication of dermal rugosities and associated scale impressions. The parietal is perforated by a prominent pineal foramen in the centre of the parietal table (figure 1). The posterior margin of the parietal is wide and gently bowed anteriorly, with an apomorphically broad nuchal fossa (electronic supplementary material, figure S2). Ventrally, a prominent pit is visible for the processus ascendens of the supraoccipital, a feature not seen in crown-gekkotans, but more commonly seen in iguanians, scincomorphans, lacertoids and anguimorphans. This distribution suggests that †*Helioscopos dickersonae* is retaining a feature that is plesiomorphic of (at least) *Squamata* that is lost further crownward within *Pan-Gekkota*. The thin supratemporal processes are slightly longer than half of the antero-posterior length of the parietal table and taper posterolaterally, ending with a slender posterior projection (electronic supplementary material, figure S2).

The maxilla of †*Helioscopos dickersonae* is typical of lizards in being anteroposteriorly longer than mediolaterally wide, and the facial process is incompletely preserved, with the right maxilla apparently preserving the entire suborbital ramus (electronic supplementary material, figure S1). The facial process rises at a high angle, and the lateral margin bears a row of at least three mental foramina (taphonomic damage precludes a confident assessment of the full number). Medially, the palatal shelf extends over the tooth row and would have articulated with the palatine. The suborbital ramus tapers posteriorly and would have terminated in the anterior half of the orbit (figure 1). The maxilla has spaces for roughly 20 teeth [14] with unicuspid apices that are slightly recurved. The posterior maxillary dentition appears to have a slight medial inflection [14] and is more obtusely pointed, although distinct accessory cusps cannot be discerned on the CT scan.

The prefrontal is large and robust, with a long, tapering supraorbital process (the anterior process is broken; electronic supplementary material, figure S1). Medially, the prefrontal is bowed posteriorly to form the posterior border of the nasal capsule and possesses a vertical medial margin (electronic supplementary material, figure S1), which we recover alternatively as either a synapomorphy of *Squamata* or *Scleroglossa* depending on whether a constraint based on molecular phylogenies is used during analysis of our morphological dataset (electronic supplementary material). The jugal is mediolaterally thin (potentially a taphonomic artefact) and smoothly arcuate, with a prominent maxillary ramus and a complete postorbital ramus, both of which taper distally from the taller main body of the jugal. A quadratojugal process of the jugal is absent (electronic supplementary material, figure S2), which we find to be either a synapomorphy of *Pan-Gekkota* on morphological trees or (ambiguously) as a retention of the ancestral squamate condition on molecular trees (electronic supplementary material). The squamosal is elongate, thin and gently curved posteroventrally, tapering to a point on which the head of the quadrate would pivot, characteristic of squamates (electronic supplementary material, figure S2). It lacks any suggestion of an ascending process, which we find as synapomorphic of *Pan-Gekkota* (under a molecular constraint this implies a loss from the reduced condition seen in several scincomorphs, while without constraints it is a loss from the iguanian condition).

The palatines are incompletely preserved. The choanal and maxillary processes are both broken, but an appreciable portion of the pterygoid process is preserved (electronic supplementary material, figure S4). The palatines are smooth, edentulous, and ventrally bear a choanal fossa that arches strongly over the choana (electronic supplementary material, figure S4). The right pterygoid is partially preserved, showing the confluence of the palatine, transverse and quadrate rami, but none of these processes are intact (electronic supplementary material, figure S4). The transverse ramus is the thinnest of the three rami. A ridge runs along the ventral surface of the pterygoid (electronic supplementary material, figure S4). The edentulous nature of the palatines and pterygoids of †*Helioscopos dickersonae* is shared with crown gekkotans [5] although this appears convergent as stem gekkotans crownward of †*H. dickersonae* retain at least some palatal teeth [23]. We infer that the vomers were likely edentulous, as no living or extinct squamates that lack palatine and pterygoid teeth possess vomerine teeth [24]. The partial braincase (figure 1) is mediolaterally wide and slightly dorsoventrally compressed (though this may be taphonomic), and the occipital condyle has a transversely flat posterior margin in ventral view (electronic supplementary material, figure S3).

The dentaries are dorsoventrally shallow and taper towards their anterior ends (figure 1). On the lateral surface of each dentary, there is a row of four mental foramina of roughly equal size (electronic supplementary material, figure S5). Medially, a thickened ridge underlies the positions of at least 16 unicuspid dentary teeth, of which the posterior teeth are more obtusely pointed than the anterior (electronic supplementary material, figure S5). One of the posterior teeth has a slightly tricuspid appearance but it is unclear if this represents the true tooth morphology or taphonomic damage. While 16 tooth positions are clear in the CT scan, there must have been more than 20, as in squamates the dentary always has more teeth than the maxilla (except in some scolecophidian snakes [3]). Articular facets for the splenial are present, and Meckel's canal is exposed along the length of the dentary (ventrally at the tip; electronic supplementary material, figure S5). The left prearticular and surangular are fused (= compound bone) but lack their anterior and posterior ends (figure 1). A comprehensive description of the anatomy of †*Helioscopos dickersonae* is included in the electronic supplementary material for this article.

### (c) Systematic paleontology

*Squamata* Oppel 1811 (*sensu* de Queiroz and Gauthier 2020)

*Pan-Gekkota* Bauer 2020

†*Ardeosauridae* Camp 1923 [15] (see Taxonomic Notes in electronic supplementary Information)

†*Limnoscansor* gen nov.

Type species – †*Homeosaurus digitatellus* N. M. Grier 1914

†*Eichstaettisaurus digitatellus* M. Cocude-Michel 1963

†*Ardeosaurus digitatellus* R. Hoffstetter 1964

**Etymology.** †*Limnoscansor,* derived from the Ancient Greek *limnes* for ‘lake or marsh’ denoting the lagoonal environment of the Solnhofen limestone, and *scansor* from the Latin *scansarius* ‘to climb’ after its purported climbing-adapted anatomy [25].

**Figure 2.**
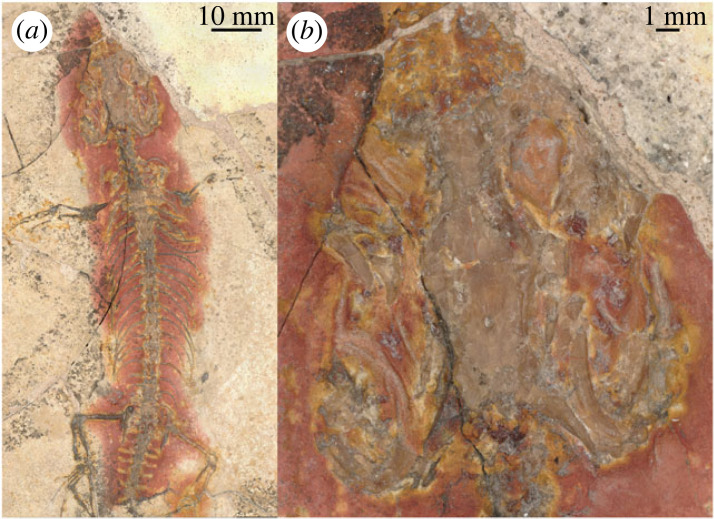
Close-up photographs of *Limnoscansor digitatellus* (CM 4026). (*a*) Full body of specimen (dorsal view). (*b*) Close up of skull (dorsal view).

**Holotype.** CM 4026, partial skeleton including the skull posterior to the snout, as well as cervical and trunk vertebrae, pectoral and pelvic girdles, fore- and hindlimbs, and the base of the tail. Specimen preserved as a slab in dorsal view (figure 2).

**Locality and Horizon.** Wintershof, Eichstätt, Bavaria. Eichstätt lithographic limestone (Solnhofen group), Tithonian (152–149Ma) (Hybonotum zone, Reidense subzone) [26].

**Comments on ontogenetic stage.** Neither the braincase nor the posterior mandibular elements are visible in CM 4026 and the specimen is not amenable to histological analysis. Thus, confident referral to an ontogenetic stage is difficult. However, the epiphyses of the long bones are all fused in CM 4026, enabling us to reject that it is a neonate and suggesting that it has reached at least 80% of its maximum size [19].

**Differential Diagnosis.** As almost none of our phylogenetic analyses recovers ‘†*Ardeosaurus*’ as monophyletic, we propose a new generic name, †*Limnoscansor,* for the taxon formerly known as †*Ardeosaurus digitatellus*. To avoid future confusion induced by potentially differing phylogenetic positions of †*L. digitatellus* as in [27], we find it prudent to propose a new genus name for this taxon (see the online supplementary material for further discussion). †*L. digitatellus* is diagnosed by the following combination of features: preorbital skull length is shorter than postorbital skull length, a lateral ridge on the squamosal, and a lack of dermal sculpturing across the skull roof. (Characters denoted by an asterisk [*] were noted by Simões *et al*. [25].) Differs from †*Ardeosaurus brevipes* in lacking skull roof ornamentation*; having a narrower space between the parietal supratemporal processes*; having an apparently blunter snout (as noted by Mateer [28] and seen in the impression of the snout in the sediment); less mediolateral constriction of the parietal; having a ridge in the lateral surface of the squamosal (as opposed to a smooth surface); the supratemporal appears broader than in †*A. brevipes* (though this could be taphonomic); having narrower frontals (relative to the frontoparietal suture); having a relatively larger pineal foramen (relative to parietal width); having a fused postorbitofrontal*; having 27 (as opposed to 23) presacral vertebrae*; differing in pedal phalangeal formula*. Differs from †*Eichstaettisaurus* in having a straight frontoparietal suture*; having wider frontals (relative to the frontoparietal suture); having a fused parietal*; having less mediolateral constriction of the parietal*; having a narrower space between the parietal supratemporal processes*; having a fused postorbitofrontal*; having a lateral ridge on the squamosal; having a shorter but wider supratemporal; having 27 (as opposed to 33) presacral vertebrae*. Differs from †*Schoenesmahl dyspepsia* in having a straight frontoparietal suture; relatively wider frontals (relative to the frontoparietal suture); having paired frontals; having a fused parietal; having a narrower space between the parietal supratemporal processes; having a fused postorbitofrontal; having a shorter (more abbreviated) snout. Differs from †*Bavarisaurus macrodactylus* in having a straight frontoparietal suture; relatively wider frontals (relative to the frontoparietal suture); more posteriorly directed supratemporal processes of the parietal (which also create difference in the orientation of the supratemporal); having 27 (as opposed 23–25) presacral vertebrae. Differs from †*Palaeolacerta bavarica* in having the pineal foramen enclosed entirely by the parietal; wider frontals (relative to the frontoparietal suture); having a fused postorbitofrontal.

See also Simões *et al*. [25] and Conrad [27] for additional features distinguishing †*L. digitatellus* from the type species of †*Ardeosaurus*, †*A. brevipes*.

**Diagnosis of genus.** As for the type species (CM 4026).

## 3. Discussion

### (a) Complex assembly of the gekkotan skull

**Figure 3.**
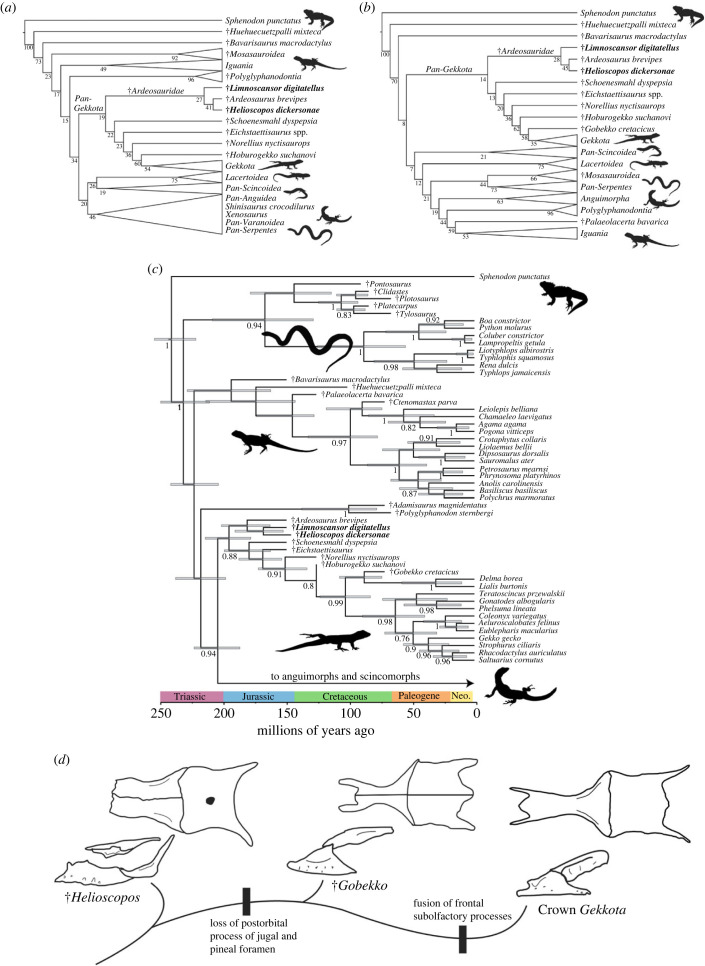
Phylogenetic position of *Helioscopos dickersonae* under different analytical regimes. (*a*) Results of unconstrained implied weights parsimony, weighting strength *K* = 12. (*b*) Results of implied weights parsimony, weighting strength *K* = 12, with the topology of the modern taxa constrained to the molecular tree of [2]. Numbers on nodes are bootstrap values (1000 pseudoreplicates). (*b*) Results of tip-dated Bayesian analysis. Numbers on nodes are posterior probabilities, while the bars represent the 95% confidence intervals of the inferred age of the node. (*d*) Reduced evolutionary tree of *Pan-Gekkota* showing several key anatomical transitions in the skull roof and face.

**Figure 4.**
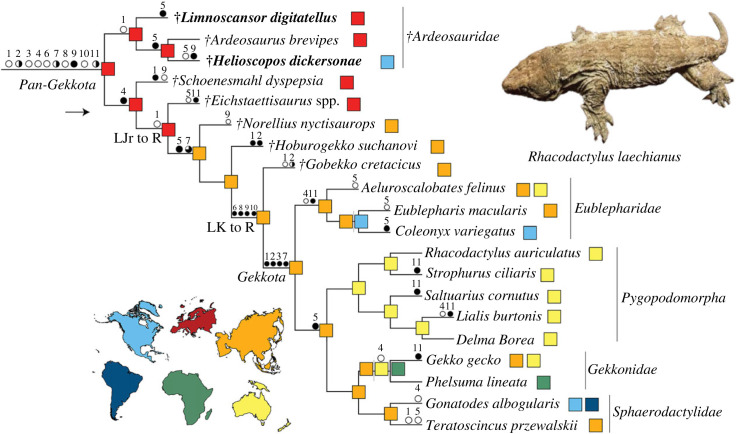
Evolution of pan-gekkotan anatomy and biogeography. Tree of *Pan-Gekkota* (from our weighted analysis *K* = 12 restricted to a molecular topology). Branch squares represent inferred continents of origin, with terminal branches based on recorded observations and internal branches being the results of our parsimony ancestral states reconstruction. Red, Europe; blue, North America; orange, Asia; green, Africa; purple, South America; yellow, Australian plate. New taxa in bold. Circles above branches represent key synapomorphies of *Gekkota* and *Pan-Gekkota* identified by [3]. Open circles represent the absence of features, closed circles represent the presence of features, and partially filled circles represent partially acquired features. 1, fusion of the frontals; 2, fusion of the frontal subolfactory processes into a tubular structure; 3, absence of a postorbital; 4, paired parietals; 5, posterior midline projection of the parietal; 6, absence of a pineal foramen; 7, prefrontal medial margin extending beneath frontal subolfactory process; 8, absence of a jugal postorbital bar; 9, edentulous palate (as gauged by the condition of the pterygoid); 10, dentary fused over Meckel's canal; 11, 31 or more maxillary and dentary teeth.

All of our analyses find †*Helioscopos dickersonae* on the gekkotan stem. Additionally, all but one of our analyses recover †*H. dickersonae* as most closely related to †*Ardeosaurus brevipes* and †*Limnoscansor digitatellus* in a clade we call †*Ardeosauridae* Camp 1923 (*sensu* [27]) (figures 3 and 4, and see electronic supplementary material). Only our analysis constrained to a molecular topology and strongly weighted (at weighting-strength *K* = 3) failed to recover †*Ardeosauridae* including †*L. digitatellus* (electronic supplementary material, figure S8). A detailed account of the phylogenetic results and full lists of relevant synapomorphies can be found in the electronic supplementary material.

We find that 11 of the 28 osteological synapomorphies of *Gekkota* identified by Gauthier *et al*. [3] first appear variably on the gekkotan stem (figure 4). Some are acquired in a stepwise fashion towards the crown. Others are more variable in appearance across *Pan-Gekkota*. For example, the medial margin of the prefrontal changes in a stepwise fashion along the pan-gekkotan stem as it acquires the crown condition, where it underlaps the frontal subolfactory process [3]. The plesiomorphic vertical orientation observed in †*Helioscopos dickersonae* (electronic supplementary material, figure S1) was supplanted by the ventromedially sloping margin of the more crownward †*Norellius nyctisaurops* (see fig. 6 of Conrad & Norell [21]), which was in turn further developed in the crown (figure 4). Additionally, †*Gobekko cretacicus* shares the absence of an ectopterygoid posterior process with *Gekkota* [22], while the more stemward taxon †*N. nyctisaurops* [21] retains a small posterior process, supporting a gradual loss of this feature in the gekkotan stem. Finally, †*H. dickersonae* and other ardeosaurids retain a discreet jugal postorbital process, which is subsequently reduced in †*G. cretacicus* and lost in more crownward pan-gekkotans (figure 3*c*).

Other characters display states incipient to those characterizing the crown but lack such a smoothly sequential transitional series. These include the fused subolfactory processes that give the gekkotan frontals a tubular appearance (figure 4). The beginnings of this transition are seen in stem gekkotans such as †*Helioscopos dickersonae* in which the processes approach one another without touching (figure 4). As in the crown, these processes are in contact in the successively more crownward stem-gekkotans †*Hoburogekko suchanovi* [29] and †*Gobekko cretacicus* [22]. However, the subolfactory processes are fused, as in the crown, in the more distal stem-species †*H. suchanovi*, but they are still unfused in †*G. cretacicus*, a stem-gekkotan more proximal to the crown [22], indicating some homoplasy in this transition.

Still other characters previously considered synapomorphic of the crown first appear variably across the gekkotan stem, but do not appear to become fixed until the emergence of *Gekkota* proper. These include the fusion of the frontals observed in *Gekkota* (except in the sphaerodactylid *Teratoscincus*), with paired frontals being retained in most stem gekkotans (including †*Helioscopos dickersonae;* figure 3*c*), but with fusion occurring in some stem species such as †*Schoenesmahl dyspepsia* [27] and †*Hoburogekko suchanovi* [29]. An absence of pterygoid dentition (indicating an edentulous palate) is shared between the gekkotan crown [7] and †*H. dickersonae*. However, species even closer to the crown, †*S. dyspepsia* [27] and †*N. nyctisaurops* [23], retain pterygoid teeth.

Some characters that show a complex distribution in the crown also show a complex distribution on the stem. These include the presence or absence of a parietal midline projection [3], which is absent in †*Helioscopos dickersonae* and †*Eichstaettisaurus* [3], but which is present in †*Limnoscansor digitatellus* (Plate XXII of Grier 1914 [30]) and †*Ardeosaurus brevipes* (Text-Fig. 2 of Mateer 1982 [28]), as well as in the more crownward †*Norellius nyctisaurops* [3] and †*Gobekko cretacicus* [31]. Additionally, paired parietals often associated with crown *Gekkota* (except eublepharids) are also seen in almost all stem gekkotans, and regardless of tree topology we recover paired parietals as ancestral to the clade stemming from the ancestor of †*Schoenesmahl dyspepsia* + *Gekkota*. However, all ardeosaurids, including †*H. dickersonae*, have fused parietals as in squamates generally, including dibamids [3] (figure 3*c*). Fusion is alternatively interpreted as either the ancestral state for *Pan-Gekkota* or the first independent evolution of this feature at the base of the gekkotan total clade (a transition that occurs at least four times within our small sample of *Gekkota;* figure 4).

Several apomorphies, such as the loss of the postorbital, still appear restricted to the gekkotan crown [3]. Nevertheless, our analysis reveals a complex history of mosaic character change on the gekkotan stem, with some characters gradually attaining their modern states and others being variably present or absent across the stem, before eventually becoming fixed in the crown.

### (b) Insight into the lifestyle of †*Helioscopos dickersonae*

†*Helioscopos dickersonae* retains a prominent pineal foramen in the parietal, particularly when compared to its close relative †*Ardeosaurus brevipes*. This feature is absent in nocturnal *Gekkota* [1], and its loss is first seen in the stem-gekkotan †*Gobekko cretacicus* [32] (figure 3*c*). It has been hypothesized that this signals the acquisition of nocturnal habits in *Pan-Gekkota* [32], as the parietal eye serves as a light-sensing organ [33]. By regulating the timing and quantity of melatonin production, it serves to track day length, thereby regulating circadian rhythms and light-dependent physiological processes such as thermoregulation [34]. As such, the presence of a pineal foramen in †*H. dickersonae* indicates that day-length tracking and light-regulated signalling were still important to its physiology, meaning it was neither fully nocturnal nor fossorial. Additionally, the presence of this feature in †*Schoenesmahl dyspepsia,* which was found as the gut contents of the dinosaur †*Compsognathus longipes* [27], may imply diurnal or crepuscular foraging in the latter species.

### (c) Pan-gekkotan phylogeny tracks patterns of Jurassic biogeography

**Figure 5.**
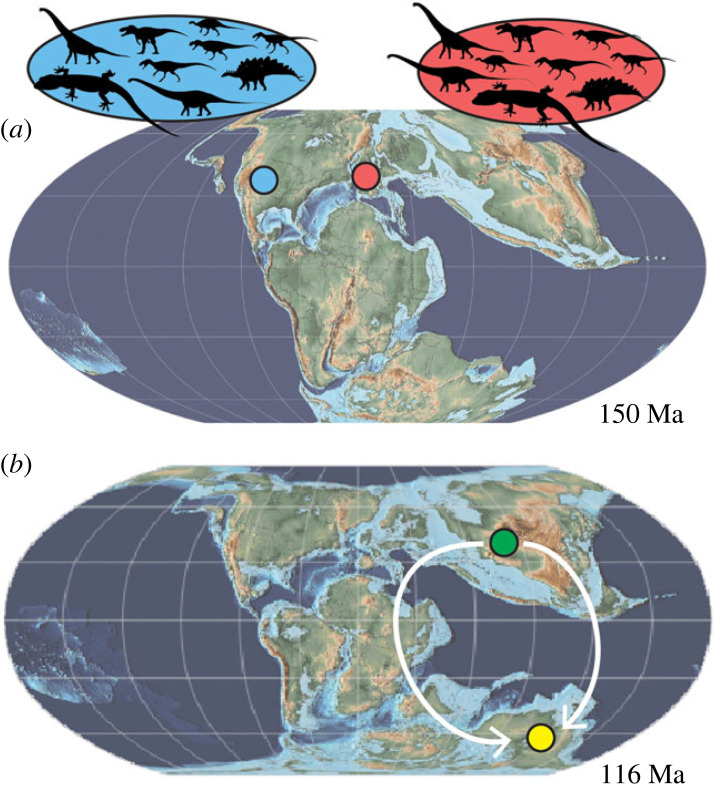
Historical biogeography of *Pan-Gekkota*. (*a*) The distribution of sister taxa found at European and western North American sites. The red dot indicates the hypothesized European origin of *Pan-Gekkota* and the blue dot represents the previously unknown North American radiation. (*b*) The phylogeographic hypothesis for pygopodoids generated in this study. The green dot represents the estimated Asian origin of *Gekkota* and the yellow dot represents the estimated Australian origin of *Pygopodoidea*. White lines represent the two possible scenarios of pan-pygopodoid dispersal out of Asia supported by this study. Paleogeographic reconstructions constructed with GPlates.

There is a striking similarity between the Late Jurassic dinosaur faunas of North America and Europe. Numerous iconic dinosaur taxa, including †*Allosaurus* [35], †*Torvosaurus* [36], †*Ceratosaurus* [35], †*Supersaurus* [37] and †*Stegosaurus* [38] are present in penecontemporaneous deposits in both the North American Morrison and Portuguese Lourinhã Formations (figure 5). The discovery of the North American species †*Helioscopos dickersonae* and its close relationship to †*Ardeosaurus brevipes* and †*Limnoscansor digitatellus* of the German Solnhofen Limestone establishes another broadly distributed Laurasian clade of Jurassic squamates, alongside the North American paramacellodids [24], the Morrison specimens referred to the (possible) pan-anguimorph †*Dorsetisaurus* [39], and to †*Diablophis gilmorei* [40] which was originally referred to the English taxon †*Parviraptor* [41]. †*H. dickersonae* illustrates that faunal similarities across the Late Jurassic Northern Hemisphere extended across multiple size, ecological and physiological classes of terrestrial vertebrates. It also confirms the extension of the known zone of squamate continuity between the English Purbeck Formation and Portuguese Lourinhã and Alcobaça formations far to the west in the USA, as noted by [14,39,40], and further extends this eastward to the German Solnhofen archipelago.

It is unsurprising that similarity is seen among Jurassic–Cretaceous dinosaur faunas across Laurasia given their large body sizes and elevated metabolic rates [42]. These traits would have enabled dinosaurs to occupy larger home ranges than those of small, ectothermic squamates, as home-range size correlates positively with both body size [43] and metabolic scope [44]. The recognition of a North American–European clade of Jurassic pan-gekkotans establishes that there was a degree of environmental homogeneity across these regions conducive to squamate dispersal across smaller home ranges. This observation is consistent with the generally similar paleolatitude of North America and Europe during the Jurassic (figure 5*a*).

The North American provenance of †*Helioscopos dickersonae* also illuminates the early biogeography of pan-gekkotans and contributes to a growing synthesis of the origins of the wide distributions of various gekkotan backbone clades today. The imperfect and heterogeneously sampled nature of the fossil record renders firm conclusions about historical biogeography in deep time problematic. Nevertheless, our analyses illustrate the importance of considering the fossil record when interrogating such questions.

Incorporating both continental- and hemispheric-locality data into parsimony ancestral state reconstructions based on our constrained and unconstrained analyses supports a Laurasian origin of *Pan-Gekkota*, with western Eurasia (Europe) being the most parsimonious region of origin (figures 4 and 5). The most parsimonious region of origin for gekkotans crownward of †*Eichstaettisaurus* is eastern Eurasia (Asia) (figures 4 and 5). This result is congruent with a European origin for the gekkotan total clade in the Jurassic, followed by dispersals west into North America and east into Asia, and the subsequent origin of the crown among Late Cretaceous squamate assemblages in Asia (figure 5). Considering only the extant representatives of *Gekkota,* it would be equally parsimonious for the clade to have originated in Asia or Southern Gondwana (including modern Australia), regardless of the favoured topology. Additionally, fossils such as †*Helioscopos dickersonae* illuminate a previously unknown entry of stem gekkotans into the USA during the Jurassic and their apparent extinction there, followed by a reinvasion of the crown roughly 100 million years later [45].

Initial hypotheses of gekkotan biogeography invoked vicariance through the breakup of Pangea [46] and early molecular-clock estimates supported this idea; Jonniaux & Kumazawa [47] for example, proposed a crown divergence in the mid-Jurassic. Subsequent molecular analyses have, however, inferred a much more recent initial divergence, either in the Early Cretaceous (144 Ma [48]) or most recently in the mid-Cretaceous (approx. 126 Ma [2]), both of which are more compatible with the fossil record and our phylogenetic analysis, which supports a mid-Cretaceous divergence of *Gekkota* (figure 3*c*). Although most gekkotan backbone clades are geographically widespread today, our fossil-record-conditioned optimization of continental biogeography supports an Asian origin for the crown (figure 4). That is to say, Asia appears to be the ancestral setting for the probable earliest-diverging crown-gekkotan backbone clade, the *Eublepharidae* (figure 4; [2,45]), as well as the later-diverging clade composed of *Sphaerodactylidae* + *Gekkonidae* (figure 4), all of which have descendants still living there today. This interpretation is compatible with the previously proposed western-Asian origin of *Sphaerodactylidae* [49], followed by subsequent circum-Mediterranean spread and long-distance, transoceanic dispersals from western North Africa [45], an island for much of this time [50], to the Americas bordering the western margins of Tethys (figure 5).

**The endemic Australian gekkotans here called *Pygopodoidea*** [15] refers to the crown-clade composed of more ‘snake-like’ *Pygopodidae* and more ‘lizard-like’ *Carphodactylidae* and *Diplodactylidae*. Pygopodoids, being so modified, have posed long-standing challenges phylogenetically and biogeographically. Both morphology (e.g. [46]) and molecules (e.g. [2]) now agree that pan-pygopodoids (the pygopodoid total clade) represent the second crown-gekkotan backbone divergence within *Gekkota*. But that still leaves open the question of how crown pygopodoids arrived in Australia from a stem rooted in Asia? There are two contrasting biogeographic histories consistent with the current understanding of gekkotan phylogeny. The first scenario involves a Neogene dispersal out of mainland southeast Asia into Australia. This pattern characterizes many other Australian squamates, but they all have close relatives in mainland Asia, which is not the case for pygopodoids [49]. Moreover, the basal divergence within crown pygopodoids significantly antedates those of other Australian squamate clades. Estimated divergence times for the pygopodoid crown in Australia range from approximately 70 [49] to approximately 57 [2] million years ago, when that continent was still far from Asia across the Indian Ocean. But the total clade *Pan-Pygopodoidea* is much older (approx. 116 Ma, Aptian [2]; figure 3*c*), when a conjoined Australia + Antarctica (=Australarctica) was still near East-Gondwanan land masses [51]. Our data are more compatible with the second proposal of pan-pygopodoid biogeography [49], in which this clade first dispersed from Asia across Tethys into eastern Africa, then travelled overland through Africa to again disperse overwater onto Australarctica, where the crown subsequently originated by the Early Eocene. This scenario is not exclusive of additional spread into South America via Africa, or a more complicated southward spread of *Pan-Pygopodoidea*, but represents the minimum steps of a southward dispersal of the clade. That said, it is difficult to choose among these alternatives when there are no unambiguous pan-pygopodoids, either Recent or fossil, known from anywhere in the world apart from Australia (and some nearby islands) [10]. Whichever biogeographic history eventually prevails, it is nevertheless clear that the landmasses of the Australian plate serve as a refuge for a once far more widespread gekkotan backbone clade, just as they have for the monotreme mammals, perichelydian turtles and the last of the rhynchocephalian lepidosaurs.

## 4. Methods

### (a) Photography

The holotype of †*Limnoscansor digitatellus* was photographed at high magnification using a Keyence VHX-7000 digital microscope (Keyence, Itasca, IL, USA).

### (b) Computed tomography

DINO 15914 was scanned by Jessie Maisano on 29 September 2008 at the University of Texas High-Resolution X-ray CT Facility. The scan parameters were: 180 kV, 0.13 µA, no filter, 1400 projections, 2 frames per projection. Digital segmentation of the individual elements was conducted in 3D Slicer [52] and VGStudio MAX 3.5.

### (c) Phylogenetic dataset

To assess the phylogenetic affinity of *Helioscopos dickersonae* within *Squamata*, we used a modified version of the dataset of Gauthier *et al*. [3], one of the largest and most comprehensive morphological analyses of squamates. We increased the sampling of known and proposed stem gekkotans and followed the character modifications of Brownstein *et al*. [24] (including incorporating characters from Longrich *et al*. [53]) and additionally revised several characters in the dataset. For a complete list of modifications to the matrix see the online electronic supplementary material.

### (d) Parsimony analyses

We performed multiple analyses under parsimony using the program TNT v. 1.5 [54]. Characters were treated as ordered following their status in Gauthier *et al*. [3] (see online electronic supplementary material for a full list of ordered characters). For all runs, *Sphenodon punctatus* was selected as the outgroup. An initial analysis was performed under equal-weights parsimony using the new technology search function. Sectorial, ratchet, drift and tree fusing were all used under default parameters except for changing the number of ratchet iterations to 100. We performed a driven search using the default parameters but modifying the initial random-addition sequences to 50 and finding the minimum length 10 times. Following the new technology search, we performed a second round of tree bisection–reconnection (TBR) via traditional search on the trees held in RAM. To assess node support, we performed bootstrap resampling tests with 1000 pseudoreplicates on the consensus trees of our parsimony analyses. These were performed using new technology searches (sectorial, ratchet [100 iterations], drift and tree fusing), with five initial random-addition sequences and finding the minimum length one time.

As weighted parsimony has been proposed to be more powerful than equal weights parsimony for morphological datasets [55], we also performed analyses using the implied weights setting in TNT. We began by using the recommended *K* value of 12 [55] for our weighted analyses (figure 2.) and tested the sensitivity of our results by running additional analyses under *K* values of 3, 6 and 24 (electronic supplementary material, figures S8 and S9). Implied weights analyses were performed under the same protocol as the equal-weights analyses apart from modifying the new technology search from finding the minimum length to stabilizing consensus (with default parameters).

We also performed analyses constrained to the molecular topology of Burbrink *et al*. [2]. We constrained the extant taxa to their positions in the molecular tree and designated the fossil taxa as floaters. We performed both equal- and implied-weights analyses (electronic supplementary material, figures S7, S8 and S9) of the constrained dataset following the previously outlined protocol.

#### (i) Bayesian phylogenetic analysis

As a further test of the affinities of †*Helioscopos dickersonae* to major squamate clades, we conducted a tip-dated Bayesian analysis on a modified version of our morphological dataset using the Fossilized Birth-Death Model as implemented in BEAST 2.6.6. [56,57]. Because of the computational load required to conduct Bayesian tip-dating analyses on large morphological datasets with numerous high-state-count characters, we reduced the dataset to include a subset of 96 of the taxa included in our original morphological matrix. These taxa represent the most complete representatives of major fossil clades, as well as representatives of all major living clades except the problematic long-bodied, limb-reduced *Dibamidae*, *Amphisbaenia*, *Anniella* and *Pseudopus.* We used the Markov-variable model of character evolution presented in [58] and partitioned characters by state count. We used a loose gamma prior with default values for the mean and standard deviation and a lognormal relaxed clock. The origin prior was set following [24], and the outgroup was set as †*Sphenodon punctatus* using a monophyletic MRCA prior. The diversification rate was set as 0.27, which is the proportion of living species in the dataset divided by the mean origin prior time, which is 251.9 Ma. We ran the analysis twice independently over 2.0 × 10^8^ generations with 1.0 × 10^8^ pre-burnin. Convergence of the posteriors was checked using Tracer 1.7.1 [59] and the resulting posterior tree sets were combined in LogCombiner v. 2.6.7 and summarized in TreeAnnotator v. 2.6.4 [56].

#### (ii) Ancestral state reconstructions

Ancestral state reconstructions were performed using Mesquite v. 3.70 [60] under the parsimony ancestral states method. To estimate the biogeographic history of pan-gekkotans, we added two unordered characters to our phylogenetic matrix, one for continent of origin (either Europe, Africa, Asia, North America, South America or Australia) and one for hemisphere of origin (either North or South). We then used parsimony ancestral states in Mesquite to estimate the point of origin of various nodes.

#### (iii) Nomenclatural acts

The nomenclatural acts contained in this publication have been registered in ZooBank. The LSID for this publication is urn:lsid:zoobank.org:pub:DE35E794-8E08-47E8-958C-B95D48467187. The LSID for genus *Helioscopos* is urn:lsid:zoobank.org:act:6075D640-DCE3-41C0-BA20-55D51AB18FE0. The LSID for the species *Helioscopos dickersonae* is urn:lsid:zoobank.org:act:23C94ECD-6571-4E57-A605-952853A12275. The LSID for the genus *Limnoscansor* is urn:lsid:zoobank.org:act:471B19A8-0EA0-41D4-B537-5FCF1A536B96.

## Data Availability

Electronic supplementary material is available online for this paper [61] as well as files (including 3D stls of the individual elements of *Helioscopos dickersonae*, the phylogenetic matrix used in this analysis and tree files from our analyses) that can be found on Dryad at https://doi.org/10.5061/dryad.rbnzs7hjc [62].
